# Deregulated bile acids may drive hepatocellular carcinoma metastasis by inducing an immunosuppressive microenvironment

**DOI:** 10.3389/fonc.2022.1033145

**Published:** 2022-10-21

**Authors:** Jin-kun Xia, Ning Tang, Xing-yu Wu, Hao-zhen Ren

**Affiliations:** ^1^ Department of Hepatobiliary Surgery, The Affiliated Drum Tower Hospital of Nanjing University Medical School, Nanjing, China; ^2^ Institute of Hepatobiliary Surgery, Nanjing University, Nanjing, China

**Keywords:** bile acids, hepatocellular carcinoma, metastasis, gut microbiota, immunosuppressive microenvironment

## Abstract

Bile acids (BAs) are physiological detergents that can not only promote the digestion and absorption of lipids, but also may be a potential carcinogen. The accumulation of BAs in the body can lead to cholestatic liver cirrhosis and even liver cancer. Recently, studies demonstrated that BAs are highly accumulated in metastatic lymph nodes, but not in normal healthy lymph nodes or primary tumors. Lymph node metastasis is second only to hematogenous metastasis in liver cancer metastasis, and the survival and prognosis of hepatocellular carcinoma (HCC) patients with lymph node metastasis are significantly worse than those without lymph node metastasis. Meanwhile, component of BAs was found to significantly enhance the invasive potential of HCC cells. However, it is still poorly understood how deregulated BAs fuel the metastasis process of liver cancer. The tumor microenvironment is a complex cellular ecosystem that evolves with and supports tumor cells during their malignant transformation and metastasis progression. Aberrant BAs metabolism were found to modulate tumor immune microenvironment by preventing natural killer T (NKT) cells recruitment and increasing M2-like tumor-associated macrophages (TAMs) polarization, thus facilitate tumor immune escape and HCC development. Based on these available evidence, we hypothesize that a combination of genetic and epigenetic factors in cancerous liver tissue inhibits the uptake and stimulates the synthesis of BAs by the liver, and excess BAs further promote liver carcinogenesis and HCC metastasis by inducing immunosuppressive microenvironment.

## Introduction

Liver cancer is one of the common digestive system malignant tumors, and its incidence is increasing year by year (1). The most common histologic type of liver cancer is hepatocellular carcinoma (HCC), which is usually diagnosed with a poor prognosis, especially at advanced stages owing to the high rate of recurrence and metastasis (2). Although considerable progress and advances in the treatments in recent decades, including interventional therapy, curative resection, liver transplantation, targeted therapy, and immunotherapy, prognosis of HCC remains far from satisfying (3). The 5-year survival rate of advanced HCC patients remains poor generally because of the propensity for metastasis (4). Therefore, it is urgent to further elucidate the molecular pathogenesis of HCC metastasis in order to develop novel therapeutic treatments and improve the survival rate of this malignancy.

Bile acids (BAs) are a general term for a class of amphiphilic molecules produced by cholesterol metabolism, and their functions involve lipid digestion, absorption and hormone synthesis (5). In the human liver, cholesterol is metabolized to primary BAs, including cholic acid (CA) and chenodeoxycholic acid (CDCA), and then enters the gut for further conversion to the corresponding secondary BAs (6). CA and CDCA undergo biotransformation such as dissociation, epimerization, oxidation and 7α dehydroxylation activity by intestinal bacteria in the colorectum and hydrolases to generate secondary BAs such as deoxycholic acid (DCA) and lithocholic acid (LCA) (7). The secondary BAs can also be conjugated to taurine or glycine to form conjugated BAs. Secondary BAs are highly “aggressive” and toxic, thereby changes in BAs composition are the main cause of dysregulated BAs-related liver disease (8). About 95% of the secondary BAs are reabsorbed by the intestine, return to the liver through the portal vein, and then pass through the biliary tract together with the newly synthesized conjugated BAs, which is called the hepatoenteral circulation of BAs (9). In the physiological state, the above process is subject to a sophisticated regulation of a series of positive and negative feedback mechanisms. For example, the nuclear farnesoid X receptor (FXR) and membrane Takeda G protein-coupled receptor 5 (TGR5) have high affinity for BAs, and these receptors coordinate with each other to maintain BAs homeostasis (10). The expression level of FXR is down-regulated, and the methylation level of TGR5 promoter is up-regulated in HCC, revealing the complex role of the genetic and epigenetic alterations in HCC environment caused by bile acid metabolism disorder. The liver has a high-efficiency scavenging effect to maintain the concentration of BAs at a low level, so the content of BAs in peripheral blood plasma is very small. The determination of BAs in plasma can reflect the synthesis, uptake and secretion functions of hepatocytes. Therefore, alterations in BAs composition and distribution can be biomarkers and prognostic indicators for liver disease (11).

The tumor immune microenvironment (TIME) refers to the microenvironment related to immunological components such as T cells, tumor-associated macrophages (TAMs), myeloid-derived suppressor (MDSCs), and NK cells within tumors. TIME can be divided into three types, namely the infiltrated-excluded (I-E) TIMEs, infiltrated-inflamed (I-I) TIMEs, and tertiary lymphoid structures (TLSs) TIMEs according to recent human and mouse data. For detailed classification and explanation, please refer to (12). TIME is an important part of the tumor microenvironment. Cancer development and progression is influenced by the immune cell composition in TIME and controlled by the host immune system. Most tumors undergo a transition from a controllable inflammatory response to an uncontrollable inflammatory response, thereby generating a microenvironment suitable for their growth to promote tumor proliferation and invasion, metastasis, angiogenesis and immune escape (13). The proportion of different immune cells in TIME and certain immune system-related biomarkers can be used for cancer detection, prognosis and evaluation of treatment response (14). In addition, TIME also contains a variety of potential cancer therapeutic targets, such as CTLA-4 and PD-1/PD-L1, and other immune checkpoint blockade-related targets are the current hot spots for tumor targeted therapy (15). Meanwhile, crosstalk between cancer cells and immune cells in TIME creates an environment that promotes tumor growth and metastasis, which is important for tumor initiation and progression (16). For example, in TIME, the interaction between the ligand PDL1 on the surface of tumor cells and the PD-1 receptor on the surface of T cells enables tumor cell immunosuppressive signals to be transmitted to the interior of T cells, inhibiting the immune function of T cells, thereby preventing the immune system attacks tumor cells, resulting in immune escape (17). Exploring the regulatory mechanisms of the TIME not only deepens our understanding of tumor cells behavior, but also has the potential to provide new insights into drugs that promote immune checkpoint inhibitors-related anti-tumor therapy.

The disruption of local immune homeostasis is an important reason for the development of many cancers, including HCC (18). It’s reported that a variety of immune cells express BAs receptors, and the disorder of BAs balance affects the differentiation and function of various immune cells, thereby affecting the occurrence and development of HCC (19). Recently, researchers observed an interesting phenomenon: BAs are highly accumulated in metastatic lymph nodes, but not normal healthy lymph nodes or primary tumors, which brought us with some new questions: (1) What drives BAs accumulation in metastatic lymph nodes? Are the BAs produced by the lymph node metastases themselves, or from circulating sources? (2) Does high accumulation of BAs in metastatic lymph nodes accelerate liver cancer metastasis, and if so, what is its mechanism? (3) Do accumulated BAs exert some kind of ‘signal’ to regulate the TIME, thereby evading immune surveillance and promoting HCC metastasis?

## Our hypothesis

**Figure 1:**
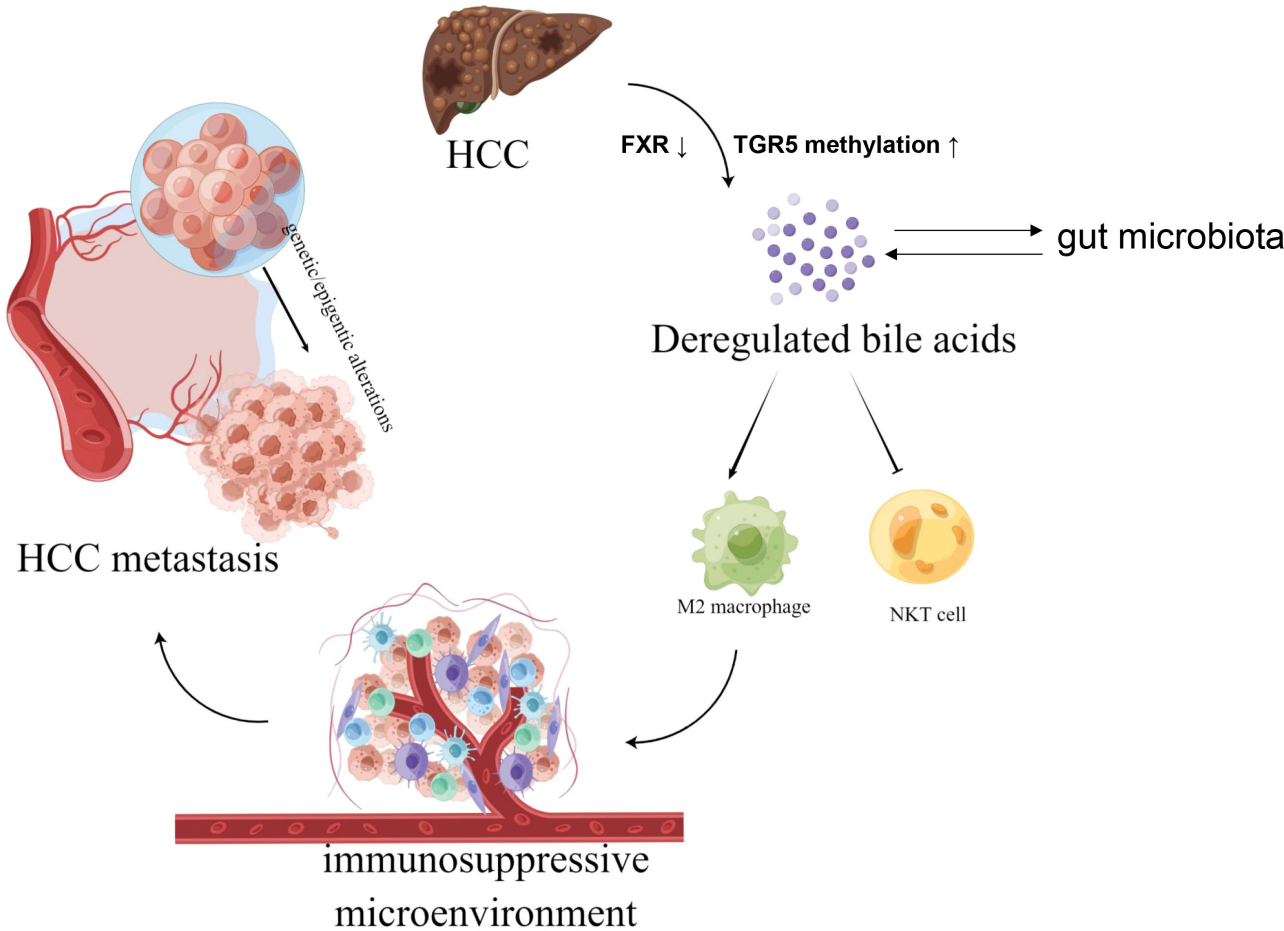
The graphical hypothesis of our study produced by FigDraw. Aberrant BAs metabolism in hepatocellular carcinoma (HCC) modulate tumor immune microenvironment by preventing natural killer T (NKT) cells recruitment and increasing M2-like tumor-associated macrophages (TAMs) polarization, thus facilitate liver carcinogenesis and HCC metastasis by inducing immunosuppressive microenvironment.

We proposed that a combination of genetic and epigenetic factors, including gut microbiota, epigenetic regulation, hepatocyte function, metabolic reprogramming, and more, in cancerous liver tissue inhibits the uptake and stimulates the synthesis of BAs by the liver, and excess BAs further promote liver carcinogenesis and HCC metastasis by inducing immunosuppressive microenvironment (**Figure 1**).

## Justification of hypothesis

### BAs are highly accumulated in HCC

BAs produced in the liver are metabolized by enzymes from gut bacteria and are essential for maintaining a healthy gut microbiota, balanced lipid, carbohydrate metabolism, and innate immunity (20). The production, transport, metabolism and excretion of BAs are carefully regulated by the body. The determination of BAs in plasma can reflect the synthesis, uptake and secretion functions of hepatocytes (21). Studies found that BAs homeostasis was disrupted during the occurrence and development of HCC (22). Serum BAs concentrations were significantly elevated in patients with HCC compared with normal subjects, thus persistence of elevated serum total BAs is considered an independent risk factor of some HCC patients (23). Elevated serum BAs may be mainly caused by leakage of damaged hepatocytes or changes in the activity of BAs transfer proteins, not necessarily by up-regulation of BAs synthesis. Elevated levels of BAs in the blood may further aggravate liver damage due to their cytotoxicity, and at the same time act as signaling molecules to promote the formation of liver tumors (24). Genetic environment and other factors cause bile outflow disorder, which can lead to cholestasis, resulting in a severe increase in serum and intrahepatic BAs levels, especially chenodeoxycholic acid levels, and these patients have a higher risk of developing liver cancer in childhood (25). During cholestasis, high concentrations of BAs can generate reactive oxygen species, damage cell membranes, damage mitochondria, and lead to DNA mutations (26). Therefore, the repair response of liver inflammation and damage caused by toxic BAs may promote tumorigenesis.

## Regulations of BAs in HCC

BAs receptors play an important role in regulating BAs synthesis, metabolism and transport. There is a high intracellular BAs load during cholestasis, and the main detoxification pathway is to activate BAs receptors to protect liver cells and attenuate BAs toxicity (27). Disorders of nuclear receptor regulation and genetic variation may promote the occurrence and development of liver diseases, including HCC (28). BAs regulate the physiological functions of cholangiocytes and hepatocytes by binding to the nuclear receptor FXR or the cell membrane receptor TGR5. They have high affinity with BAs and can regulate various physiological functions of cells. The primary BAs CDCA and CA were the most potent endogenous ligands of FXR, while the secondary BAs LCA and DCA were the most potent endogenous agonists of TGR5, respectively (10). FXR has shown its protective effect on hepatocytes in many aspects (27): (1) It can reduce the accumulation of BAs in the liver, and can also inhibit the metabolic disorder in the liver, preventing BAs from becoming a potential activator of liver cancer. Mechanistically, after FXR is activated by BAs in the intestine, it promotes the expression and release of fibroblast growth factor 19 (FGF19), and FGF19 circulates back to the liver and binds to FGF receptor 4 (FGFR4) on the surface of hepatocytes, inhibiting the rate-limiting rate of BAs synthesis expression of the enzyme cholesterol 7α-hydroxylase to maintain the balance of BAs in the body. (2) It is a negative regulator of liver inflammation and plays a key role in protecting the liver and inhibiting the occurrence of HCC. (3) By inhibiting hepatic stellate cell (HSC) aggregation, promoting extracellular matrix (ECM) degradation, preventing the occurrence and development of liver fibrosis and tumors. Indeed, FXR expression in human HCC was down-regulated compared with normal liver tissues, and FXR(-/-) mice provide a unique animal model for HCC study (29). TGR5 indirectly prevents the development of liver cancer by mainly improving metabolic syndrome; of course, TGR5 can also negatively regulate the transcriptional activity of NFκB factor, preventing chronic hepatitis disease (30). Interestingly, hypermethylation of the TGR5 promoter occurred significantly more frequent in HCC, and TGR5-/- mice have higher expression of interstitial metalloproteinases (MMPs), which may promote the development and metastasis of HCC (31, 32). This makes BAs receptors a target for the treatment of liver diseases such as cholestasis, viral hepatitis, liver fibrosis and liver cancer. At present, the FXR agonist obeticholic acid phase III clinical trial results have significant efficacy, and other FXR agonists such as GSK2324 and WAY-362450 have also entered clinical trials and achieved good clinical results (33). Targets for TGR5 are also expected to enter clinical trials in the near future.

BAs are critical components of the gastrointestinal tract that link the gut microbiota to hepatic and intestinal metabolism, and thus influence gastrointestinal motility, intestinal permeability, and carcinogenesis (34). Studies have found that intestinal flora can affect the size and composition of BAs pools. For example, Clostridium can affect the metabolism of primary BAs into secondary BAs (35). Primary BAs can increase the expression of CXCL16 in hepatic sinusoidal endothelial cells and promote natural killing in the liver. The accumulation of NKT cells enhances the anti-tumor effect of the liver; while secondary BAs have the opposite effect (36). In addition, altering the gut microbiota increased deoxycholic acid (DCA) levels; however blocking DCA production and reducing gut microbiota effectively prevented the development of liver cancer in mice (37). Apcmin/+ mice treated with deoxycholic acid (DCA) could increase the F/B value of the intestinal flora and change the composition of the intestinal flora (38), suggesting that BAs can alter the composition of the gut microbiota. BAs can also chelate with important ions such as calcium ions and ferrous ions of bacteria, which can affect bacterial gene expression and inhibit bacterial movement, reproduction and chemotaxis (39). Taken together, the distribution of BAs and gut microbiota interact with each other; BAs can modulate microbiota composition and, in turn, regulates the size and composition of BAs pools. Disruption of BAs-microbiota crosstalk promotes inflammation and many gastrointestinal disease phenotypes, which may contribute to the development of gastrointestinal cancers, including colorectal cancer and HCC.

Epigenetics refers to a group of genetic mechanisms and phenomena that do not change the genotype but can determine the phenotype of cells (40). Those heritable alterations are another way of transcriptional regulation of gene expression. Epigenetic regulation regulates posttranslational modifications (PTMs) of histones and chromatin remodeling, and thus regulates gene transcription and maintains BAs balance (41–43). Specifically, FXR acetylation is normally dynamically regulated by p300 (a histone acetyltransferase) and SIRT1 but is constitutively elevated in metabolic disease states (41). Similarly, aberrant DNA methylation of TGR5 is increased in HCC, and it can be used as a potential biomarker for hepatitis B Virus associated HCC (32). In turn, BAs can also influence epigenetic regulation and thus interfere with disease progression (44). Inhibition of histone-modified transcriptional cofactors, such as histone acetylases and methylases, has emerged as a promising and effective option for the treatment of HCC (45). Research has been devoted to identifying natural and synthetic compounds that modulate the activity of nuclear and membrane BAs receptors to alter quantity and composition of BAs (46). Therefore, an interesting future research direction is to use these compounds in combination to modulate multiple BAs signaling pathways, together with epigenetic targeting of post-translational modifications of histones or chromatin remodeling of BAs target genes to develop more effective preventive and therapeutic treatments with HCC associated with imbalances in BAs metabolism.

## BAs contribute to HCC metastasis

There are a large number of blood vessels and lymphatic vessels in the liver that can transport cancer cells to distant organs, and subsequently the metastatic tumor cells implant in sites with suitable environment to form new tumors (the ‘seed and soil’ hypothesis proposed by Stephen Paget), which limit the curative effect and prognosis of HCC patients (47). Cancer foci releases tumor cells into the bloodstream, which can be transferred to different parts inside and outside the liver with the bloodstream. The common organ of distant metastasis is the lung, and it can also be transferred to the bone, adrenal gland and brain (48).

BAs play an increasingly prominent role in cancers invasion and metastasis, although the mechanism of action seems unclear. In this regard, BAs are the most widely studied in gastrointestinal cancer and have been shown to drive multiple signaling pathways to enhance the invasiveness and metastatic ability of tumor cells (49). Several studies have shown that more hydrophobic BAs as LCA, DCA and CDCA, are the main promoters of liver cancer and promotes the motility of HCC cells by inducing EMT phenotypes (50, 51). Unlike the direct effect on cancer cells, Lu Gao et al. found that Glycochenodeoxycholate (GCDC), an important component of BAs, promotes HCC invasion and migration by AMPK/mTOR dependent autophagy activation (52). This phenomenon could be reversed by inhibition of autophagy. Meanwhile, DCA induced HSCs to secrete senescence-associated secretory phenotype (SASP) factors, thereby indirectly promoting the invasion/migration of liver cancer cells (53). Moreover, there is increasing evidence that high levels of BAs are highly clinically relevant in patients with highly aggressive HCC (52). In addition, several chemical drugs (such as The FXR Agonist Obeticholic Acid and Bushen Jianpi) can inhibit liver tumor metastasis in orthotopic mouse xenograft model and reduce the recurrence and metastasis rate of HCC patients by interfering with BAs levels (**Table 1**) (56, 57). Although information is limited, BAs also play a role in promoting HCC angiogenesis to a certain extent, that provides good conditions for tumor metastasis (59). In summary, BAs drive the progression of liver tumor metastasis in an indirect way, and antagonizing excessive BAs may provide an effective means of treating liver cancer. It should be pointed out that BAs with different concentrations and components may lead to different liver cancer progression and outcomes (60), which requires further proof in follow-up experiments (**Table 2**).

**Table 1:** Drugs targeting dysregulated bile acids in HCC.

Drugs	Mechanism of targeting bile acid	Effects in hepatocellular carcinoma	References
cholestyramine	a bile acid sequestrant	prevents HCC development	(54, 55)
Bushen Jianpi	increases expression of the bile acid receptor	inhibits liver cancer recurrence and metastasis	(56)
Obeticholic Acid, GW4064, PX20350 and PX20606	FXR agonists	suppresses HCC proliferation and metastasis	(57, 58)

**Table 2:** Tumor metastasis promoter effects of bile acids in hepatocellular carcinoma.

Cell lines	Concentration of bile acids	Effects	References
HuH-7, Hep3B	CDCA (100 µM)	induces EMT process	(50)
Hep3B	LCA, CDCA (100 µM)	promotes the motility of HCC cells by inducing EMT phenotypes	(51)
SMMC7721, Huh7	GCDC (200 µM)	promotes HCC invasion and migration by AMPK/mTOR signaling	(52)
Huh7	DCA(80 µM)	resulted in cellular senescence in LX2 and promoted HCC cells migration and invasion	(53)

CDCA, chenodeoxycholic acid; LCA, lithocholic acid; GCDCA, Glycochenodeoxycholic acid; DCA, Deoxycholic acid.

## BAs induce suppressive TIME

Inflammation is an immune defense response gradually formed by the body to resist foreign pathogens and respond to tissue damage in the long evolutionary process, mainly manifested in vascular response, immune cell recruitment and cytokine release, and has long been associated with cancer (61). Inflammation enables the cancer hallmark-promoting programs through the tumor microenvironment (TME), and diverse cells of the TME can deliver a series of cytokines to maintain the inflammatory environment in which cancer cells survive and impair antitumor immune responses (62). Since BAs can stimulate the secretion of various cytokines and chemokines, its dysregulation has been shown to be involved in the regulation of inflammation and immunity (63). Especially in digestive system tumors, BAs-involved inflammatory regulation is associated with carcinogenesis through so-called bridging factors, including signaling pathways NFκB, COX-2, STAT3, and inflammatory factors such as IL-6, IL-1β, and TNF-α etc (49). Secondary BAs, as metabolites of intestinal flora, regulate the function of immune cells, thereby achieving host immune tolerance to commensal bacteria (64). In addition, it was found that primary BAs instead of secondary BAs can also control the recruitment of natural killer T (NKT) cells in the liver by promoting the expression of the chemokine CXCL16, thereby exerting anti-tumor immune function to inhibit the growth of liver cancer (36). Several studies have also found that DCA initially induces HSCs to produce senescence-associated secreted (SASP) factors, which in turn stimulate the secretion of pro-inflammatory and tumor-promoting factors, and ultimately lead to nonalcoholic steatohepatitis (NASH) and subsequent development of HCC (37, 53). BAs also act as a signaling mediator to stimulate their nuclear receptor and promote M2-like macrophage polarization, creating an immunosuppressive TME that favors tumor-initiating cells (TICs) (54). Accordingly, high serum levels of taurocholic acid correlate with increased M2-like tumor-associated macrophages (TAMs) in HCC patient samples. Generally, macrophages have the property to be polarized to M1 or M2 macrophages, and TAMs have been illustrated to more closely resemble the M2‐polarized macrophages, which are activated by Th2 cytokines (IL‐10, IL‐13, and IL‐4) (65). Intriguingly, an increasing body of evidence suggests that M2 macrophages can facilitate the aggressiveness of HCC in many ways (including M2 macrophage-derived exosomes, plasminogen activator inhibitor−1 pathway, imbalance of TGF-β1/BMP-7 pathways, etc.) (65–67). Recently, loss of SIRT5 (a member of deacetylating enzymes) was reported to promote bile acid-induced immunosuppressive microenvironment and hepatocarcinogenesis (54), suggesting that epigenetics may be involved. Furthermore, the performance of BAs-activated receptors (BARs) and transporters in inflammation and immune regulation has also been extensively reported. Studies found that attenuation of FXR signaling can promote liver cancer development by downregulating the function of BAs transporters, inducing BAs retention and persistent inflammation in the liver (68). Conversely, administration of cholestyramine, a BAs sequestrant, significantly inhibited the development of NASH-HCC by promoting the excretion of hydrophobic BAs (55). Although the immunoregulatory role of TGR5 in HCC has not been reported, the BAs-TGR5 signaling axis may balance the production of pro- and anti-inflammatory cytokines by regulating the polarization state of macrophages, thereby controlling subsequent gastrointestinal carcinogenesis (69). Briefly, BAs on the one hand stimulate/reduce the secretion of inflammatory factors such as IL-6 and TNF-α, thereby activating/inactivating signaling pathways associated with cancer promotion to improve/inhibit cancer growth or invasiveness. On the other hand, BAs promote the tumor immunosuppressive environment by regulating the recruitment of immune cells such as NKTs and the polarization state of macrophages, thereby controlling cancer proliferation and invasion.

## Immune regulation of metastasis

External factors of the tumor, especially stromal and immune cell populations, are equally important determinants of metastatic spread. Tumors actually prepare a supportive and receptive microenvironment for colonization for the arrival of disseminated cancer cells by inducing many systemic molecular and cellular changes, called a premetastatic niche (70). It is characterized by increased angiogenesis and vascular permeability, ECM remodeling, chronic inflammation and immunosuppression (71). Chronic inflammation in the (pre)metastatic niche is an important driver of metastasis by promoting the recruitment of bone marrow-derived cells (BMDCs) and tumor cells to distant organs (72). Finally, the establishment of an immunosuppressive microenvironment is an essential feature of (pre)metastatic niches. It allows cancer cells to evade immune recognition and develop large metastases (73).

## Enrichment analysis of BAs-activated receptors

**Figure 2:**
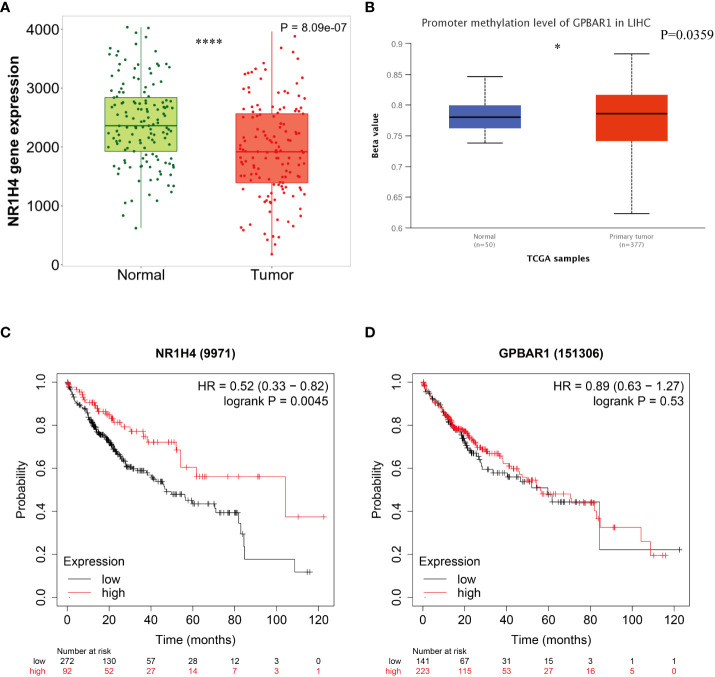
Survival curves according to the expression of bile acids- activated receptors. **(A)** Comparative analysis of mRNA expression of FXR between HCC tissue and normal tissues. **(B)** Promoter methylation level of TGR5 in HCC. Kaplan-Meier curves for overall survival according to the expression of FXR **(C)** and TGR5 **(D)**. *P < 0.05 ;**** P < 0.0001.

**Figure 3:**
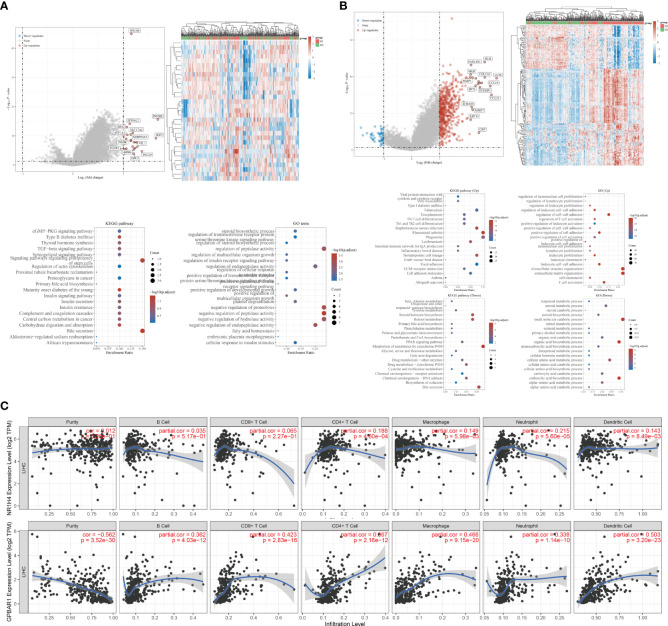
Potential biological mechanisms and immune infiltration levels of bile acids- activated receptors. **(A)** Volcano map of DEGs, heatmap of DEGs, and the Gene Ontology (GO) and Kyoto Encyclopedia of Genes and Genomes (KEGG) analyses for high and low FXR expression group based on the TCGA dataset. **(B)** Volcano map of DEGs, heatmap of DEGs, and the Gene Ontology (GO) and Kyoto Encyclopedia of Genes and Genomes (KEGG) analyses for high and low TGR5 expression group based on the TCGA dataset. **(C)** Correlations between FXR, TGR5 and immune infiltration levels validated with the TIMER database, respectively.

BAs receptors coordinate with each other to keep BAs levels relatively stable, regulate BAs-mediated signal transduction pathways, and form an effective defense mechanism to inhibit the carcinogenicity of BAs. Although FXR antagonists have been shown to inhibit HCC metastasis (58), the specific mechanism by which BAs receptors regulate HCC metastasis remains unclear. We note that FXR expression is down regulated in HCC and is associated with poor prognosis of HCC (**Figure 2**). Although the expression of TGR5 has no difference between normal liver tissues and tumor tissues, its methylation level may be involved. To reveal the potential regulatory mechanism of BAs receptors during HCC metastasis, we downloaded the transcriptome data and corresponding clinical information of FXR and TGR5 from the TCGA database, respectively, and performed enrichment analysis as described before (74). Interestingly, we found stronger evidence for TGR5 in regulating immune cell activation and cells adhesion than FXR (**Figures 3A** **, B**), albeit lacking experimental support. The expression of TGR5 is closely related to the regulation of cell-cell adhesion, T cell activation, leukocyte proliferation, etc. which are involved in the immune regulation and cell migration. In addition, TGR5 seems to regulate lymphocyte proliferation, which may explain the phenomenon of bile acid enrichment in metastatic lymph nodes. We further explored the correlation of FXR and TGR5 expression levels with immune infiltration level of immune cells in HCC using TIMER platform, respectively (**Figure 3C**). We found that the expression level of FXR correlated with the presence of CD4+T cells, macrophages, neutrophils and dendritic cells; the expression level of TGR5 correlated with the infiltration level of B-cells, CD8+T cells, CD4+T cells, macrophages, neutrophils and dendritic cells. Those results highlighted the important immune regulation of BARs in HCC.

## Discussions

Tumor metastasis and recurrence are important reasons that hinder the clinical benefit of HCC patients. Sorafenib is the first-line clinical drug for the treatment of liver cancer, but recent studies have pointed out that instead of hindering HCC migration, it further promotes tumor metastasis (75). Therefore, clarifying the mechanism of tumor metastasis has important guiding significance for the search for effective clinical drugs.

In recent years, immunotherapy, alone and in combination with conventional therapy, has achieved relatively satisfactory results in clinical trials. However, we still lack a scientific understanding of the immune-related mechanisms that influence metastasis formation and treatment response. The local and systemic immune landscapes of different types of tumors, and even individual tumors of the same type, vary widely (76). This not only reduces the specificity of immunotherapy, but also has the potential to induce multiple adverse drug reactions. Therefore, we reasoned that searching for the origin of the induced immunosuppressive microenvironment during HCC metastasis might provide an opportunity for this question. Circulating pathways among different organs and circulating components may be important reasons for the heterogeneity of tumor metastasis in different organs. BAs link various organs of the digestive tract through its enterohepatic circulation, and exploring the mechanism of action of BAs in HCC metastasis will advance the understanding of tumor metastasis heterogeneity. Additionally, administration of BA sequestrants or FXR agonists significantly prevented HCC development, suggesting a potential strategy of targeting dysregulated BAs levels for the treatment of patients with HCC, especially those with abnormally high BAs (54, 55). BAs metabolism has been shown to be highly correlated with tumor metastasis and immune regulation, but evidence linking them is still lacking. By combining literature evidence and bioinformatics analysis, we infer that the disturbance of BAs balance caused by BAs receptor dysfunction may promote the progression of HCC metastasis by inducing an immunosuppressive microenvironment. In this process, gut microbiota and epigenetic regulation may play a key role. On the one hand, gut microbiota imbalance leads to abnormal BAs metabolism, which in turn affects its immune regulation, including the recruitment of NKTs and the polarization of macrophages, resulting in the formation of an immunosuppressive microenvironment. On the other hand, epigenetic regulation promotes the occurrence of immunosuppressive microenvironment in a multi-level and multi-dimensional way, including but not limited to: regulation of BARs, cytokines, stromal cells and immune cells, metabolism alteration and pre-metastatic niche. Our study revealed cross-talk between BAs and infiltration of tumors by immune cells, and their influences in HCC metastasis, which may provide novel insight into immunotherapy of HCC. Nevertheless, more animal experiments and clinical studies are still needed to validate our hypothesis.

## Data availability statement

The original contributions presented in the study are included in the article/**Supplementary Material**. Further inquiries can be directed to the corresponding authors.

## Author contributions

J-KX and NT systemically analyzed the feasibility of the scientific hypothesis and wrote the paper. H-ZR and X-YW designed and constructed the scientific idea. All authors contributed to the article and approved the submitted version.

## Funding

This work was funded by the National Natural Science Foundation of China (82270646), the Fundamental Research Funds for the Central Universities (0214-14380510), the Nanjing health science and technology development project for Distinguished Young Scholars (JQX19002), fundings for Clinical Trials from the Affiliated Drum Tower Hospital, Medical School of Nanjing University (2022-LCYJ-PY-35), the Chen Xiao-ping Foundation for the Development of Science and Technology of Hubei Province, China (CXPJJH121001-2021073).

## Acknowledgments

The authors would like to acknowledge the technical assistance provided by the staff of the Department of Hepatobiliary Surgery, The Affiliated Drum Tower Hospital of Nanjing University Medical School, Nanjing, China. The figure of the graphical representation was generated by Figdraw (www.figdraw.com).

## Conflict of interest

The authors declare that the research was conducted in the absence of any commercial or financial relationships that could be construed as a potential conflict of interest.

## Publisher’s note

All claims expressed in this article are solely those of the authors and do not necessarily represent those of their affiliated organizations, or those of the publisher, the editors and the reviewers. Any product that may be evaluated in this article, or claim that may be made by its manufacturer, is not guaranteed or endorsed by the publisher.
