# Supplementary material for: Deregulated bile acids may drive hepatocellular carcinoma metastasis by inducing an immunosuppressive microenvironment
Source: Front Oncol. 2022 Oct 21;12:1033145. doi: 10.3389/fonc.2022.1033145 (PMC9634065; doi:10.3389/fonc.2022.1033145)
Supplement: Supplementary file 1 [file DataSheet_1.docx]

**Supplementary Table 1 Information on HCC patients in FXR high and low expression groups based on TCGA database**

| Characteristic | Low expression of NR1H4 | High expression of NR1H4 | p |
| --- | --- | --- | --- |
| n | 187 | 187 |  |
| T stage, n (%) |  |  | 0.206 |
| T1 | 88 (23.7%) | 95 (25.6%) |  |
| T2 | 55 (14.8%) | 40 (10.8%) |  |
| T3 | 35 (9.4%) | 45 (12.1%) |  |
| T4 | 8 (2.2%) | 5 (1.3%) |  |
| N stage, n (%) |  |  | 0.622 |
| N0 | 127 (49.2%) | 127 (49.2%) |  |
| N1 | 1 (0.4%) | 3 (1.2%) |  |
| M stage, n (%) |  |  | 0.355 |
| M0 | 140 (51.5%) | 128 (47.1%) |  |
| M1 | 1 (0.4%) | 3 (1.1%) |  |
| Age, median (IQR) | 63 (54, 70.75) | 59 (50, 68) | 0.011 |

**Supplementary Table 2 Information on HCC patients in TGR5 high and low expression groups based on TCGA database**

| Characteristic | Low expression of GPBAR1 | High expression of GPBAR1 | p |
| --- | --- | --- | --- |
| n | 187 | 187 |  |
| T stage, n (%) |  |  | 0.681 |
| T1 | 94 (25.3%) | 89 (24%) |  |
| T2 | 51 (13.7%) | 44 (11.9%) |  |
| T3 | 36 (9.7%) | 44 (11.9%) |  |
| T4 | 6 (1.6%) | 7 (1.9%) |  |
| N stage, n (%) |  |  | 0.622 |
| N0 | 126 (48.8%) | 128 (49.6%) |  |
| N1 | 3 (1.2%) | 1 (0.4%) |  |
| M stage, n (%) |  |  | 1.000 |
| M0 | 138 (50.7%) | 130 (47.8%) |  |
| M1 | 2 (0.7%) | 2 (0.7%) |  |
| Age, median (IQR) | 63 (53, 69.5) | 61 (51, 68) | 0.161 |
